# PPIXpress and PPICompare webservers infer condition-specific and differential PPI networks

**DOI:** 10.1093/bioadv/vbaf003

**Published:** 2025-02-11

**Authors:** Hoang Thu Trang Do, Sudharshini Thangamurugan, Volkhard Helms

**Affiliations:** Center for Bioinformatics, Saarland University, Saarbrücken 66041, Germany; Center for Bioinformatics, Saarland University, Saarbrücken 66041, Germany; Center for Bioinformatics, Saarland University, Saarbrücken 66041, Germany

## Abstract

**Summary:**

We present PPIXpress and PPICompare as two webservers that enable analysis of protein–protein interaction networks (PPINs). Given a reference PPIN and user-uploaded expression data from one or multiple samples, PPIXpress constructs context-dependent PPINs based on major transcripts and high-confidence domain interactions data. To derive a differential PPIN that distinguishes two groups of contextualized PPINs, PPICompare identifies statistically significant altered interactions between multiple context-dependent PPINs from PPIXpress. We present a case study where PPIXpress and PPICompare webservers were used in combination to construct the PPINs specific for melanocytic nevi and primary melanoma cells, and to detect the rewired protein interactions between these two sample types.

**Availability and implementation:**

PPIXpress and PPICompare webservers are available at https://service.bioinformatik.uni-saarland.de/ppi-webserver/index_PPIXpress.jsp and https://service.bioinformatik.uni-saarland.de/ppi-webserver/index_PPICompare.jsp, respectively. Alternatively, the webservers and application updates can be found at https://service.bioinformatik.uni-saarland.de/ppi-webserver/.

## 1 Introduction

Protein–protein interaction networks (PPINs) play a pivotal role in organizing the proteomics landscape that controls most cellular activities. A wide range of software exist to setup such networks based on knowledge pooled across various databases. Some tools also allow to infer context-dependent interactions that contribute to the complexity of different biological systems (Hollander *et al.* 2021) and provide insights into protein rewiring events. One workflow is based on PPIXpress (Will and Helms 2016) and PPICompare (Will and Helms 2017), two software developed in our research group. For example, PPIXpress has been employed to construct tissue-specific PPINs between normal and carcinoma tissues across 13 cancer types (Kataka *et al.* 2020). So far, PPIXpress–PPICompare remains one of only two workflows that enable users to prune a protein interaction network based on transcriptomic data of their choice (Hollander *et al.* 2021, Louadi *et al.* 2021).

Here, we introduce two novel webservers for PPIXpress and PPICompare with core functionality similar to the standalone versions. Our services aim at streamlining the PPIN analysis workflow and at providing a comprehensive view of the dynamics and alterations in network topology. The PPIXpress webserver enables users to infer tissue-specific networks from a self-provided PPIN or database-available PPIN using transcript/expression data uploaded as input. In a second step, significant rewiring events are identified by PPICompare by comparing two sets of networks constructed by PPIXpress for two different conditions. Both webservers provide downloadable visualization of condition-impacted protein interactions or domain interactions.

## 2 Methods

### 2.1 PPIXpress

PPIXpress infers condition-specific PPINs from a reference PPI network based on transcript abundances in user-defined samples by performing two main tasks: protein-domain mapping and network contextualization (Will and Helms 2016). PPIXpress requires as inputs one reference protein interactions network file and at least one expression data file. In the first step, PPIXpress translates the reference PPIN into a reference domain–domain interaction network (DDIN) by mapping each pair of proteins involved in an interaction to two protein domains that have been documented to interact. This task requires domain annotations from the Pfam-A database (Mistry *et al.* 2021) and high-confidence domain interaction data from DOMINE (Yellaboina *et al.* 2011) and IDDI (Kim *et al.* 2012), supplemented by the most recent 3did/iPfam databases (Finn *et al.* 2014, Mosca *et al.* 2014). Note that, if no Pfam domain annotation exists for a protein, PPIXpress defines a dummy domain for it. Using the expression data of a specific sample, PPIXpress then prunes the reference DDIN to a network that contains only interactions between dominantly expressed transcripts. The set of PPIs explained by at least one DDI remaining after trimming is the condition-specific PPIN as the main output of PPIXpress. Users may select optional auxiliary results including a list of major transcripts and corresponding DDINs.

### 2.2 PPICompare

PPICompare identifies a set of protein interactions that are statistically significantly rewired between two groups of samples represented by condition-specific PPINs (Will and Helms 2017). First, for each inter-group pair of samples i, PPICompare defines a pairwise rewiring probability $P_{\mathrm{rewired}\_i}$ as the fraction of rewired edges (both vanishing and appearing) over all edges present in all samples. The total rewiring probability for the initial differential network Δ between the two sample groups is computed by averaging all inter-group rewiring probabilities:


$$P_{\mathrm{rewired}}=\frac{1}{N}\sum_{i}^{N}P_{\mathrm{rewired}\_i}$$

For an edge alteration (u,v) between protein nodes u and v  ((u,v)∈Δ) to be a true randomly rewired event, its occurrence must be significantly greater than the occurrence of random edges in the differential network Δ. The likeliness for (u,v) to be detected due to random variations in expression is computed by a one-tailed binomial test:


$$p(u,v)=1-\sum_{j=0}^{|\Delta (u,v)|-1}{(}_{j}^{N}){\left(P_{\mathrm{rewired}}\right)}^{j}{\left(1-P_{\mathrm{rewired}}\right)}^{N-j}$$

whereby |Δ(u,v)| is the annotated number of rewiring events of this edge over all *N* pairwise comparisons. After applying a Benjamini–Hochberg correction to the p-values, only significant rewired events are retained in the final differential network. PPICompare also identifies a minimal portion of transcripts that could generate the systemic rewiring. By viewing transcripts and their edge alterations as two classes of a bipartite graph, finding the smallest set of transcripts that explain the largest number of rewiring events becomes a weighted set cover problem. Each transcript, or rewiring reason i, is assigned a score $s_{i}$ and a weight $w_{i}$ subsequently:


$$\begin{array}{c} s_{i}=pw_{i}\times rw_{i}\\ w_{i}=s_{\max}-s_{i} \end{array}$$

where $rw_{i}$ is the number of alterations caused by reason i, $pw_{i}$ is the number of pairwise comparisons where those alterations are found, and $s_{\max}$ is the maximum value of $s_{i}$ over all considered transcripts. PPICompare solves the problem of minimizing the sum of weights using a greedy heuristic approach (Chvatal 1979). This final step results in a small set of transcriptomic changes that explain the systematic differences in the differential network between two cell conditions.

## 3 Workflow for differential protein interaction networks analysis

**Figure 1.**
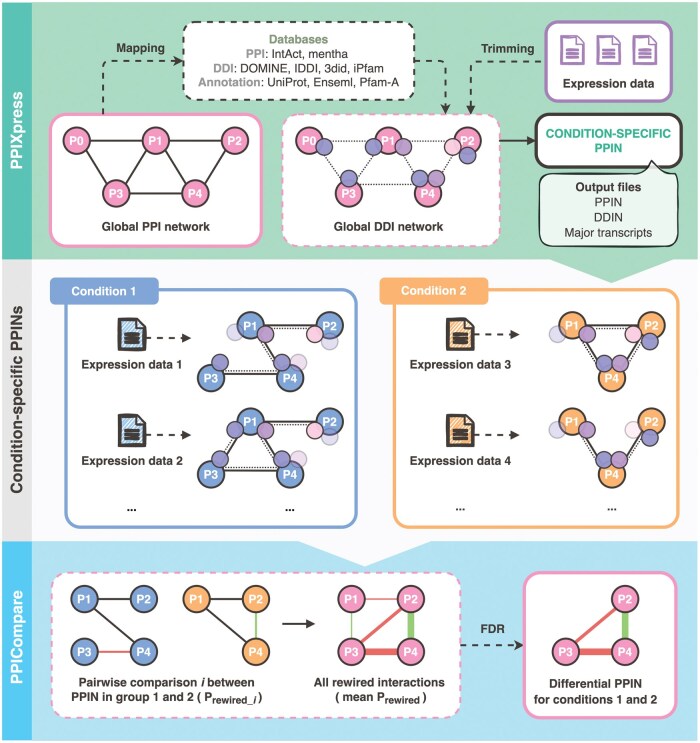
Workflow of PPIXpress and PPICompare webservers for protein network analysis. The boxes lined in solid represent input/output in the front-end that allow users to use and interact with the tools. Boxes lined in dashed include information about databases used by the tools or analysis processes in the back-end. Within a network, protein–protein interactions and domain–domain interactions are denoted by solid lines and dashed lines, respectively. In the middle panel, the faded nodes show strongly down-regulated or not expressed domains that are pruned from the global DDI network by PPIXpress when constructing the condition-specific protein–protein interaction networks.

Our differential PPIN analysis workflow comprises of PPIXpress and PPICompare webservers, which can be run independently. Figure 1 shows the workflow of a typical project.

### 3.1 PPIXpress webserver

PPIXpress analysis entails (i) data upload and setting of parameters, (ii) construction of condition-specific network(s), and (iii) query and visualization of a protein network. In part (i), PPIXpress requires two types of input, a reference PPIN, which can be defined by the user or retrieved from IntAct (Orchard *et al.* 2014) or mentha (Calderone *et al.* 2013) databases given a taxon number, and at least one user-defined transcript/expression data set. Expression data required for network contextualization can be provided in various file formats, including outputs of popular transcript alignment and quantification tools (such as Cufflinks, GENCODE, Kallisto, RSEM, and TCGA RSEM), or plain text formats with either FPKM (Fragments Per Kilobase Million) or TPM (Transcripts Per Kilobase Million) values. The construction of condition-specific network(s) (ii) is carried out for each uploaded expression data set, presumably representing different tissues or experimental conditions. Using the set of most abundant genes or transcripts of a protein from a sample, PPXpress derives condition-specific DDIs which dictate if a DDI in the original PPIN should be retained. The remaining DDIs are mapped back to the reference PPIN, resulting in condition-specific PPIs.

In the visualization panel of the PPIXpress webserver, the user may query a protein to inspect its direct neighbors in the PPIN that was pruned using the expression data from a particular tissue or condition. The display of the DDIN underlying this PPIN can be toggled by expanding or collapsing a protein node. Currently, the PPIXpress webserver only allows single-protein query instead of full protein networks, as Cytoscape.js takes quite some time for rendering a large number of protein nodes, especially when domain nodes are added. Alternatively, the trimmed networks for each tissue or condition can be downloaded and used as input to a local installation of Cytoscape on the user’s end. Apart from the condition-specific networks, users can download DDINs, and the most abundant genes or transcripts used for their construction, which are essential inputs for the differential PPIN analysis of PPICompare.

The PPIXpress webserver allows enriching the reference network with functional association scores from the STRING database (Szklarczyk *et al.* 2019), ELM motifs, or updated UniProt accessions numbers for accurate domain annotation and mapping (Uniprot 2023). Data for DDI mapping can be retrieved from 3did (Mosca *et al.* 2014) as default, or from locally precompiled DOMINE (Yellaboina *et al.* 2011), IDDI (Kim *et al.* 2012), and iPfam (Finn *et al.* 2014) data sets. While users can use and prioritize gene abundance over transcript abundance for DDIN construction, PPIXpress also allows the normalization of transcript-level expression data by transcript lengths. The threshold for a gene or transcript to be considered expressed can be adjusted in the input of PPIXpress webserver. Its optimal value may depend on the particular application scenario. The PPIXpress analysis can be further tuned by removal of nonsense-mediated decay transcripts. A Help page and FAQs section provide a detailed list of input requirements and execution options for PPIXpress, help with the interpretation of its output, and explain how PPIXpress results may be transferred to the PPICompare webserver.

### 3.2 PPICompare webserver

**Figure 2.**
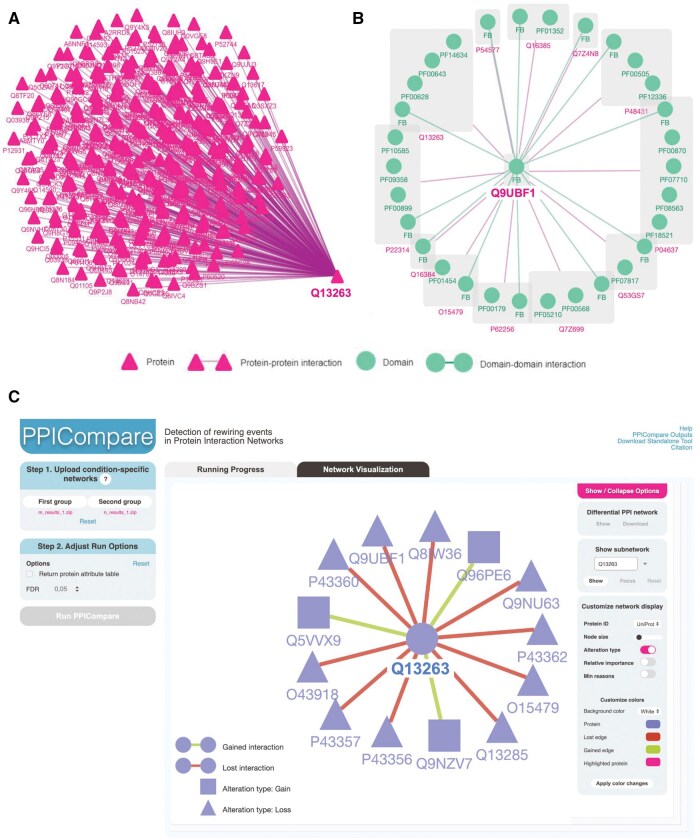
Case study of analysing the differential protein–protein interaction network (PPIN) between melanocytic nevi and primary melanoma samples by PPIXpress and PPICompare webservers. (A and B) Two examples for the network visualization by the PPIXpress webserver, where the PPINs specific for BO-027-SM_M2 sample from the dataset GSE112509 containing the proteins Q13263 (TRIM28) and Q9UBF1 (MAGEC2) are shown, respectively. (C) A snapshot of a differential PPIN constructed by PPICompare that encapsulates the differences between nevi- and melanoma-specific PPINs from PPIXpress. Here, gained and lost interactions in nevi samples are, respectively, denoted by green and red edges, while the corresponding emerging proteins are represented by triangle nodes and vanishing proteins by square nodes.

The PPICompare webserver performs differential analysis on multiple condition-specific PPINs generated, e.g. by PPIXpress, including (i) data upload, (ii) detection of significantly rewired interactions and transcriptomic changes responsible for such changes, and (iii) visualization of altered protein interactions. Two datasets are required for PPICompare, each must contain three types of outputs from PPIXpress, namely the condition-specific DDINs (*_ddin.txt(.gz)), PPINs (*_ppin.txt(.gz)), and a list of major genes or transcripts (*_major_transcripts.txt(.gz)). In the core part (ii), PPICompare compares the interactomes between the uploaded conditions in a pairwise manner and derives the set of inter-group rewired interactions with a probability for each alteration. The false discovery rate (FDR) is set by default to 0.05 as a threshold for significant rewiring interactions. Figure 2 showcases the network of proteins involved in the transcriptomic alterations between the compared conditions, together with the lost and gained interactions between them.

## 4 Case study

The PPIXpress webserver was used to construct condition-specific PPINs for RNA-seq datasets of primary melanoma samples and melanocytic nevi (Gene Expression Omnibus series ID GSE112509) (Kunz *et al.* 2018). To retrieve the reference human interactome, the taxon ID 9606 was provided. The gene expression data for each sample was loaded with the options set to “Gene-level only” and the expression level threshold set to 1. The run options for “Output DDINs” and “Output major transcripts” were selected as they are required for further processing using PPICompare. The condition-specific networks for each sample were constructed and visualised using their corresponding protein IDs. As two examples, Fig. 2A and B show the interactors of proteins Q13263 and Q9UBF1 identified in a melanoma sample. A complete list of the interactors of Q13263 and Q9UBF1 in each sample of the melanoma and nevi groups is provided in Supplementary Material S1.

The primary melanoma- and melanocytic nevi-specific networks were loaded as two input groups into PPICompare. Then, we selected the option to return the attribute table and set the FDR for significantly altered PPIs to 0.05. The significant rewiring events between the two groups were constructed as a differential network and visualised in the “Network Visualisation” panel (shown in Fig. 2C). The analysis found that the interaction between the proteins Q13263 (gene name: TRIM28) and Q9UBF1 (gene name: MAGEC2) is significantly rewired between those two groups. This is consistent with previous studies (Song *et al.* 2018, Liu *et al.* 2022) showing that MAGEC2 interacts with TRIM28 in melanoma cells. In tumour cells, the expression levels of MAGEC2 depend on the expression levels of TRIM28, as a significant decrease in MAGE28 levels was observed in TRIM28 depleted cells.

To our knowledge (Hollander *et al.* 2021), there exists no tool comparable to PPICompare that detects interactome rewiring events and the according transcriptomic alteration for user-defined datasets. SPECTRA (Micale *et al.* 2015) and DifferentialNet (Basha *et al.* 2018) are two webservers that also generate context-specific differential PPINs, but both allow only the analysis of human data. There are several similar tools to PPIXpress; nonetheless, they have different focuses, such as predicting alternative isoforms (DIIP (Ghadie *et al.* 2017)) or DDI-based missing interactions at isoform/exon-level (DIGGER (Louadi *et al.* 2021)).

## 5 Conclusion

Two new webservers PPIXpress-web and PPICompare-web are introduced that enable biomedical scientists to identify statistically significant rewirings between PPINs of two conditions. The established workflow supports rapid inference of protein networks directly from gene or transcript expression data with network visualization and protein query in constructed condition-specific or differential networks.

## Supplementary Material

vbaf003_Supplementary_Data
